# Application of Natural Products in Neurodegenerative Diseases by Intranasal Administration: A Review

**DOI:** 10.3390/pharmaceutics17050675

**Published:** 2025-05-20

**Authors:** Yu Jin, Xinyu Ma, Shuo Liu, Shiyu Zong, Yunlong Cheng, Hong Zhang, Chunliu Wang, Ye Li

**Affiliations:** 1 Pharmacy College, Shaanxi University of Chinese Medicine, Xianyang 712046, China; jy8505@email.sntcm.edu.cn (Y.J.);; 2 Key Laboratory of TCM Drug Delivery, Shaanxi Academy of Traditional Chinese Medicine, Xi’an 710001, China; 3 Institute of Traditional Chinese Medicine, Shaanxi Academy of Traditional Chinese Medicine, Xi’an 710001, China

**Keywords:** intranasal administration, natural products, neurodegenerative diseases, Alzheimer’s disease, Parkinson’s disease, Huntington’s disease

## Abstract

Natural products derived from traditional Chinese medicine have received significant attention as potential treatments for neurodegenerative disorders due to their wide availability, demonstrated efficacy, and favorable safety profiles. Intranasal delivery provides distinct advantages for targeting the central nervous system (CNS), enabling direct therapeutic agent delivery to the brain by bypassing the blood-brain barrier (BBB). This review evaluates natural products administered intranasally for neurodegenerative diseases (NDs), highlighting their therapeutic potential and addressing formulation challenges related to physicochemical properties. Strategic optimization approaches are proposed, including novel carrier systems, molecular modifications, and combination therapies. By discussing current difficulties and offering practical recommendations, this review aims to encourage further scholarly research and clinical application.

## 1. Introduction

Neurodegenerative diseases (NDs) comprise a group of disorders characterized by a progressive decline in cognitive, memory, and motor functions resulting from selective neuronal loss [1]. The pathogenesis of NDs primarily involves genetic factors, oxidative stress, mitochondrial dysfunction, cellular autophagy, and immune inflammation [2]. Clinically, NDs are categorized into two groups based on pathological manifestations. The first group includes diseases with cognitive impairment and memory loss, such as Alzheimer’s disease (AD), vascular dementia, frontotemporal dementia, mixed dementia, and Lewy body dementia. The second category affects the motor system and includes amyotrophic lateral sclerosis, Huntington’s disease (HD), Parkinson’s disease (PD), multiple sclerosis, and spinal cerebellar ataxia [3]. The escalating prevalence of NDs, particularly in aging populations, has emerged as a pressing global health challenge, profoundly impacting patients’ quality of life and imposing substantial socioeconomic burdens. The etiology, age of onset, and characteristic clinical manifestations of neurodegenerative diseases are shown in Table 1.

The precise etiology of NDs remains unclear, and there is currently a lack of effective therapeutic options. Hence, the pursuit of safe and efficacious therapeutic interventions has emerged as a focal area in neuroscience research. In this context, natural products offer unique advantages, such as simultaneously targeting multiple tissues and molecular pathways, reducing toxicity and side effects, and overcoming drug resistance [11]. Studies have demonstrated that natural products can modify the brain microenvironment, act on cellular signaling pathways, promote nerve regeneration, inhibit neuronal apoptosis, and enhance neural stem cell proliferation [12]. Moreover, significant progress has been made in exploring the therapeutic potential of natural products for NDs. Consequently, greater attention should be directed towards enhancing the efficacy of natural products in the treatment of NDs.

Current therapeutic strategies for NDs primarily involve oral administration as the main peripheral delivery route. However, this method is limited by low bioavailability caused by hepatic first-pass metabolism, gastrointestinal degradation, and systemic clearance [13]. Injectable drug delivery also faces poor brain bioavailability due to high plasma protein binding and the restrictive nature of the blood-brain barrier (BBB). Current data indicate that over 98% of small molecules and virtually all macromolecules cannot penetrate the BBB effectively to reach the brain [14]. Consequently, efficient targeting of drugs to the brain remains a significant challenge in ND therapeutics. Recent advancements have explored intrathecal injection as a direct drug delivery method to the central nervous system (CNS). However, this invasive route can cause pain, discomfort, nausea, headache, neurotoxicity, and other adverse effects [15]. Compared to peripheral administration, intranasal administration (IN) represents a non-invasive alternative characterized by rapid onset, improved patient compliance, and avoidance of hepatic first-pass metabolism. Additionally, this route facilitates direct drug absorption through the olfactory and respiratory mucosa, enabling CNS delivery via dual transport pathways: intra-neuronal and extra-neuronal mechanisms. Thus, it effectively bypasses the BBB, potentially mitigating adverse effects associated with systemic drug absorption and making it a promising route for delivering ND treatments [16].

In summary, utilizing natural products via IN for NDs treatments capitalizes on the broad availability, defined components, and multi-target activity of these compounds. It also combines the advantages of IN delivery, such as rapid onset and ease of use. Nevertheless, translating natural products into clinically viable nasal formulations presents several challenges. These include intrinsic limitations of natural compounds, such as poor aqueous solubility, potential mucosal irritation, cytotoxicity, restricted drug loading capacity, poor mucosal adhesion, and mucociliary clearance. Researchers have proposed valuable experimental solutions to these problems; however, these strategies have not yet been comprehensively summarized. In this paper, we consolidate recent progress in using natural products administered intranasally for NDs treatments. We also discuss current challenges and feasible solutions to provide valuable guidance for future research on IN delivery of natural products for NDs treatments.

## 2. NDs

### 2.1. AD

AD is the most prevalent neurodegenerative disease. Globally, the prevalence of AD is approximately 5–7%. Among individuals aged 65–69 years, the incidence of AD is estimated to be 500–700 cases per 100,000 population. This incidence significantly increases in populations over 85 years of age, reaching 30,000–50,000 cases per 100,000 individuals [17]. The pathogenesis of AD remains incompletely understood. It is characterized by amyloid plaque deposition from Aβ aggregation and neurofibrillary tangles resulting from tau protein hyperphosphorylation. Both processes cause neuronal damage and glial cell accumulation [18]. Cholinergic damage has been hypothesized to contribute to AD development. Acetylcholine is closely linked to human cognitive function. The absence of central cholinergic neurotransmitters in AD patients leads to disorientation, memory loss, and behavioral and personality changes [19,20,21]. Furthermore, oxidative stress, inflammatory responses, gene mutations, and other factors have been proposed as contributing to AD pathogenesis [22].

Currently, drug therapy remains the primary treatment for NDs. It can relieve symptoms but cannot delay or halt the progression of brain tissue lesions. For example, therapeutic drugs for AD include acetylcholinesterase inhibitors (donepezil hydrochloride, carbadine) and non-competitive NMDA receptor antagonists (memantine). These drugs may improve cerebral metabolism, circulation, and provide neuroprotection [23]. Among these, acetylcholinesterase inhibitors are considered the most effective. However, they can induce adverse effects, including gastrointestinal disturbances and bradycardia. Additionally, these medications only provide symptomatic relief without modifying the underlying pathological mechanisms [24].

### 2.2. PD

PD is the second most common neurodegenerative disorder after AD. According to *the Global Burden of Disease Study* and extensive epidemiological data, the global prevalence of PD is estimated at 100 to 300 cases per 100,000 individuals [25]. The prevalence of PD is about 0.3% in the general population, 1% in individuals over 60 years, and 3% in those over 80 years [26]. With increasing population aging, it is projected that by 2030, the number of PD patients in China may surge to 4.9 million, constituting 57% of the global PD population [27]. This increase presents a considerable challenge to China’s healthcare system. The pathogenesis and progression of PD are complex and incompletely understood, involving the degeneration of nigrostriatal dopaminergic neurons, striatal dopamine depletion, and α-synuclein accumulation. The disease involves multiple pathways and mechanisms. In addition to established age and gender factors, lifestyle and environmental influences also play important roles. Studies have indicated interactions among oxidative stress, impaired autophagy, neuroinflammation, apoptosis, ferroptosis, abnormal neurotransmitter secretion, and abnormal accumulation of excitatory amino acids. These interactions collectively contribute to disease onset and progression [28,29].

Levodopa remains the primary therapeutic agent for PD. It functions as a dopamine prodrug that alleviates motor symptoms through conversion to dopamine. However, with disease progression and prolonged drug use, patients may experience motor complications, such as levodopa-induced anisotropy and symptom fluctuations. Furthermore, low brain uptake, poor oral bioavailability, and rapid peripheral metabolism by aromatic amino acid decarboxylase further limit clinical drug use [30,31].

### 2.3. HD

HD is the predominant single-gene disorder among NDs and exhibits an autosomal dominant inheritance pattern [32]. The global incidence of HD is approximately 0.005–0.01%, corresponding to 5–10 individuals per 100,000 population [33]. The disease primarily causes degenerative atrophy of striatal neurons, with symptoms progressively worsening over time. Most patients exhibit movement disorders, cognitive decline, and mental disturbances. Survival typically ranges from 10 to 20 years, with motor function completely lost before death [34]. HD results from the cytosine-adenine-guanine trinucleotide repeat amplification within the first exon of the human huntingtin (HTT) gene. Mutant HTT containing extended polyglutamine (poly Q) is widely expressed in brain and peripheral tissues, causing selective neurodegeneration, particularly prominent in the striatum and cerebral cortex [35,36]. The main pathological manifestations of HD involve abnormalities in neurotransmitter delivery, notably dopamine, γ-aminobutyric acid, or glutamate, with dopamine abnormalities being the most common [37].

Current therapeutic strategies for HD primarily focus on alleviating motor dysfunction, psychiatric manifestations, and cognitive decline. Tetrabenazine, the sole FDA-approved pharmacological treatment for HD, reduces choreiform movements through dopamine depletion. However, its use is limited by adverse effects, including depressive symptoms and sedation [38].

### 2.4. Other NDs

Other NDs encompass amyotrophic lateral sclerosis (ALS), spinal muscular atrophy (SMA), hereditary spastic paraplegia (HSP), and multiple sclerosis (MS). ALS is characterized by selective motor neuron loss in the spinal cord, brainstem, and motor cortex. This degeneration leads to paralysis and early mortality [39]. SMA is the most common hereditary disease in children. It is an autosomal recessive disorder caused by loss of motor neurons due to a deficiency in survival motor neuron protein [40]. HSP is a genetic disorder characterized by weakness and spasticity in the lower limbs. MS is an immune-mediated disorder defined by inflammatory myelin loss in the CNS. Lesions primarily involve white matter and cause neurological dysfunction [41,42]. As these other NDs have not been specifically reported in relation to the topic of this paper, they will not be further discussed.

## 3. Pathway of Drug Delivery via IN

Nasal administration has a long-standing tradition in China. As early as the Eastern Han Dynasty, Zhang Zhongjing’s *Synopsis of the Golden Chamber* recorded the treatment of “*Jiu Zu Si Fang: Mashing the Xie juice, Irrigation of the Nose*”, establishing a precedent for nasal emergency treatment [43]. In the 1980s, research on nasal administration for CNS diseases gained traction globally. Jensen et al. demonstrated the therapeutic effects of intranasal L-vasopressin in PD patients, pioneering IN for NDs [44]. Additionally, Vaka and Murthy showed that intranasal peppermint oil could circumvent the BBB and enhance the brain bioavailability of nerve growth factor [45]. Their findings further support the potential of nasal administration for delivering natural products in NDs.

**Figure 1:**
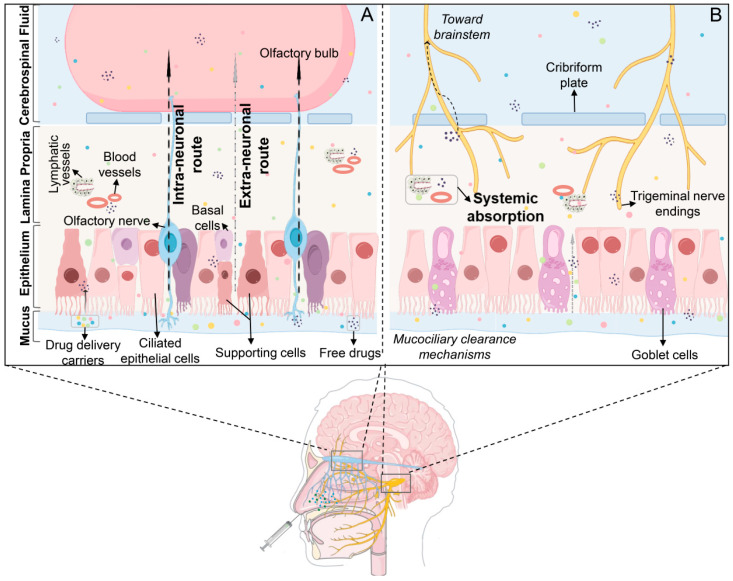
Distribution pathways of drugs administered intranasally. (**A**) Olfactory region; (**B**) Respiratory region. Following IN, drugs interact with nasal mucosa, and some are cleared to the nasopharynx via the mucociliary system on the nasal mucosal surface. The remainder enters the brain primarily through three routes: (i) Trigeminal nerve pathway: drugs are transported from nasal mucosa to the respiratory epithelium and then to the brainstem. (ii) Olfactory pathway: drugs traverse from nasal mucosa via the olfactory nerve to the olfactory bulb and subsequently the brain. (iii) Systemic pathway: drugs enter systemic circulation and cross into cerebrospinal fluid (CSF) to reach the brain.

In 1989, Erdo et al. initially suggested that medications could be directly delivered from the nasal cavity to the brain [46], although the exact transport mechanism was unclear. Currently, three primary nasal-to-brain drug delivery routes are identified: the trigeminal, olfactory, and extra-neuronal pathways (the trigeminal and olfactory routes are collectively referred to as intra-neuronal pathways) [47] (Figure 1). Following IN, drug molecules initially contact the nasal mucosa in the olfactory and respiratory regions. Some of the drugs move to the nasopharynx, being eliminated via mucociliary clearance mechanisms [48]. The remaining portion traverses the mucus layer in the respiratory region, penetrates the lamina propria through intercellular spaces, and enters lymphatic vessels or capillaries to reach systemic circulation (extra-neuronal pathway). Alternatively, drugs may be absorbed by trigeminal nerve terminals and transported along axons to the brainstem (trigeminal nerve pathway). Drugs transported via the trigeminal nerve predominantly distribute to regions such as the midbrain, pons, and hypothalamus. Unlike the trigeminal nerve, olfactory dendrites can traverse the mucosal surface, allowing drug internalization by olfactory neurons through endocytosis. These molecules then diffuse along gaps between olfactory ensheathing cells and olfactory nerve fibers, crossing the cribriform plate into the subarachnoid space (olfactory pathway). From here, they either spread directly to the brain or reach the olfactory bulb and subsequently enter the brain. Alternatively, they may be cleared by cerebrospinal fluid into lymphatic vessels, ultimately reaching the brain via systemic circulation. Drugs via the olfactory pathway primarily distribute in the olfactory bulb, anterior olfactory nucleus, frontal cortex, and hippocampus [49,50].

## 4. IN of Natural Products for NDs Treatment

### 4.1. Phenols and Phenolic Acids

**Figure 2:**
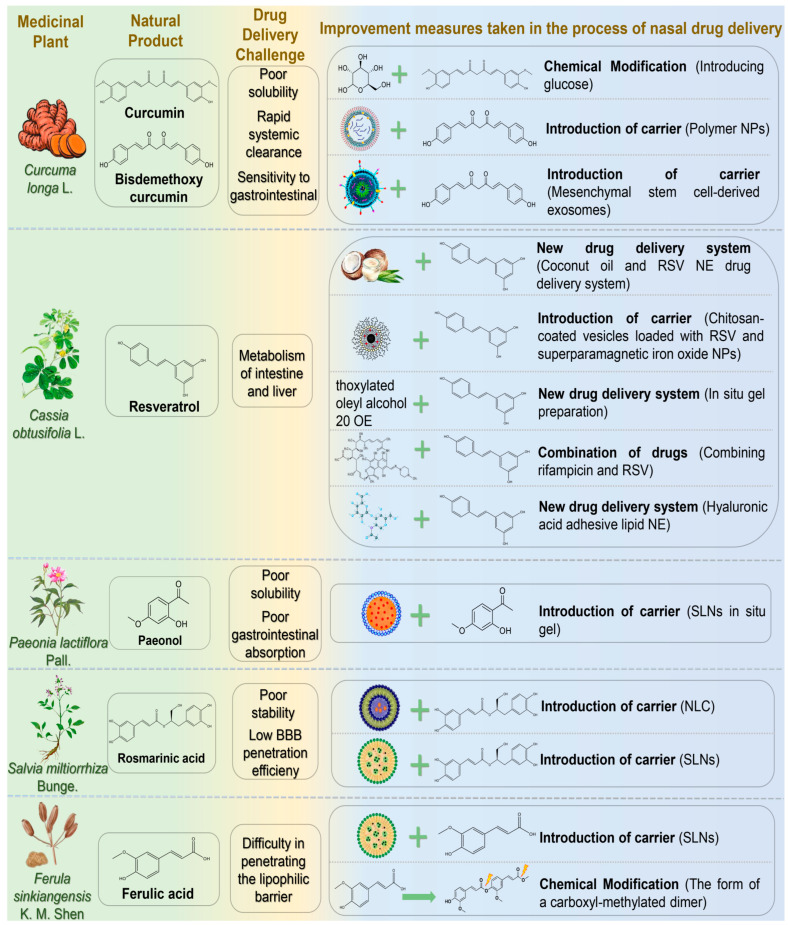
The challenges associated with the treatment of neurodegenerative diseases via nasal administration of phenolic and phenolic acid natural products and the schematic diagram illustrating potential solutions. Abbreviations: NPs, nanoparticles; RSV, resveratrol; NE, nanoemulsion; SLNs, solid lipid nanoparticles; NLC, nanostructured lipid carrier; BBB, blood-brain barrier.

Phenols and phenolic acids, widely distributed in plant species, have emerged as extensively studied natural compounds for intranasal delivery in NDs research (Figure 2) (Table 2). Their multifunctional properties include antioxidant, anti-inflammatory, and neuroprotective activities [51]. Current research is summarized below.

#### 4.1.1. Curcumin

Curcumin (CUR), first extracted from the dried rhizome of *Curcuma longa* L. (Zingiberaceae), is the main active substance of turmeric. Studies revealed that CUR possesses anti-inflammatory, antioxidant, and antiviral properties. It is used to treat several diseases involving inflammation, oxidative stress, microbial infections, cancer, and NDs [52]. Curcumin is a potent neuroprotective agent that prevents AD through multi-target molecular mechanisms [53]. First, curcumin interacts with Aβ via its phenolic hydroxyl group and β-diketone moiety, effectively inhibiting Aβ oligomerization and fibrosis, thus reducing amyloid plaque deposition. Second, it activates the Nrf2/ARE signaling pathway, enhancing expression of antioxidant enzymes, such as SOD and GSH-Px, to alleviate oxidative stress. Regarding tau protein regulation, curcumin inhibits GSK-3β activity and tau hyperphosphorylation, reducing neurofibrillary tangle formation. Although CUR exhibits substantial pharmacological activity, its clinical application in AD treatment faces challenges, including poor solubility, rapid systemic clearance, and instability in the gastrointestinal environment [54]. Additionally, intravenous (IV) administration for encephalopathy, a common clinical route, faces difficulties crossing the BBB, cell membranes, and lysosomal membranes, significantly limiting drug accumulation at target sites. IN, however, offers an efficient and convenient drug delivery alternative. Ahmadi et al. demonstrated that synthesizing curcumin glycosides from CUR by fusion reaction with glucose improved intranasal brain delivery [55]. The glycoside notably elevated glutathione and acetylcholine levels, positively contributing to spatial memory recovery in scopolamine-induced AD model rats. In another study, Hathout et al. developed polymer nanoparticles with hydrophobic properties similar to bisdemethoxycurcumin as intranasal drug carriers [56]. This drug delivery system exploited hydrophobic characteristics to enhance penetration of olfactory and trigeminal nerves for targeted brain entry. Additionally, nanoparticle interactions with nasal mucins extended the drug’s residence time. Separately, Peng et al. employed mesenchymal stem cell-derived exosomes as nanoscale natural carriers to enhance transmembrane drug transport and overcome poor brain accumulation [57]. These exosomes were loaded with CUR to form nasal self-directed nanoparticles, actively migrating to lesion sites. Treatment significantly improved motor behavior and coordination in PD rat models by reducing α-synuclein aggregation and modulating exosome function.

#### 4.1.2. Resveratrol

Resveratrol (RSV) is a naturally occurring polyphenolic compound known for medicinal and dietary properties. It can be extracted from cassia seeds, mulberry leaves, and other medicinal plants, serving as a key antitoxin in stilbene-containing species. RSV exhibits anti-inflammatory, antioxidant, anti-apoptotic, and immune-enhancing effects, demonstrating therapeutic advantages for neurological and cardiovascular disorders [58]. RSV modulates AD pathology by inhibiting Aβ aggregation, activating the Nrf2/ARE pathway to reduce oxidative stress, suppressing NF-κB to alleviate neuroinflammation, and regulating PI3K/Akt/GSK-3β signaling to decrease tau phosphorylation. It also enhances CREB/BDNF signaling, improves mitochondrial function, and inhibits microglial activation, collectively reducing cognitive decline in AD [59]. RSV can cross the BBB, and its oral absorption in humans reaches about 75%, mainly through interepithelial diffusion. However, extensive metabolism in the intestine and liver significantly reduces oral bioavailability to less than 1%. Increasing the dose or repeated administration does not improve bioavailability [60,61]. Thus, oral administration may not be optimal for RSV delivery. Kotta et al. developed an RSV nanoemulsion drug delivery system using coconut oil as the oil phase, enhancing lubrication, nostril healing, and anti-inflammatory effects [62]. Pluronic P107 and Cremoophor EL (1:1) served as surfactants to improve nasal absorption. This formulation significantly enhanced drug release and targeted delivery, achieving a peak brain concentration (5762.30 ± 316.9 ng/mL) two hours post-administration, double that of RSV suspension. The nanoemulsion also improved sustained and controlled release properties. Abbas et al. prepared chitosan-coated vesicles loaded with RSV and superparamagnetic iron oxide nanoparticles, incorporating them into sodium alginate/PVP wafers [63]. In vitro studies showed enhanced RSV release. Compared with conventional RSV solution, the vesicle formulation significantly improved cognitive and memory functions, reduced pro-inflammatory markers, and downregulated NF-κB and P38 expression in lipopolysaccharide-induced AD mouse models. These findings suggest that nasal administration combined with external magnetic targeting enhances RSV therapeutic efficacy. Fonseca-Santos et al. incorporated RSV into a surfactant-based nasal in situ gel with strong mucosal adhesion [64]. Pharmacological analysis indicated improved learning and memory in AD animal models and reduced neuroinflammation. Additionally, RSV may counteract adverse effects of rifampicin, providing new strategies for drug safety. Umeda et al. evaluated the transnasal co-delivery of rifampicin and RSV in mouse models of neurodegenerative dementia [65]. Results showed RSV reduced rifampicin-induced liver injury. Drug combinations can exert synergistic effects but may inherit individual delivery system limitations. Selecting appropriate carriers and delivery routes is thus crucial. Nasr employed hyaluronic acid-coated lipid nanoemulsions encapsulating RSV and curcumin [66]. This nanoemulsion improved stability, sustained antioxidant activity, and protected polyphenols from degradation. Safety and brain delivery studies demonstrated nasal mucosa safety and significantly increased polyphenol brain concentrations (7- and 9-fold increases, respectively).

#### 4.1.3. Paeonol

Paeonol (PAE) is a low molecular weight phenolic compound extracted from the dried root bark of *Paeonia lactiflora* Pall. It exhibits neuroprotective effects by mitigating oxidative stress, inflammation, and mitochondrial dysfunction [67]. Existing research demonstrated that PAE facilitates autophagosome formation and turnover via AMPK/mTOR signaling, effectively clearing Aβ and maintaining intracellular homeostasis [68]. Nevertheless, clinical applications of PAE are limited by poor water solubility, low gastrointestinal absorption, and rapid clearance [69]. Sun et al. developed nasal PAE-loaded solid lipid nanoparticle-based in situ gels, achieving sustained PAE release (64.89% ± 3.14% over 72 h) with minimal cytotoxicity [70]. Significant brain accumulation occurred, particularly in the olfactory bulb, cerebellum, and striatum, suggesting future therapeutic potential for NDs.

#### 4.1.4. Rosmarinic Acid

Rosmarinic acid (RA) is a principal active ingredient of *Salvia miltiorrhiza* Bunge. Recent studies demonstrated pharmacological activities protecting cardiovascular and cerebrovascular systems, widely applicable in NDs [71,72]. RA enhances cellular antioxidant defense by modulating the Nrf2/ARE signaling pathway, reducing oxidative stress [73]. Additionally, RA improves synaptic plasticity and cognitive function in neurodegenerative disease models through CREB/BDNF pathway activation [74]. However, as a polyphenol, RA suffers from poor stability and low BBB penetration, reducing pharmaceutical efficacy. Markova et al. developed a nanostructured lipid carrier (NLC) loaded with RA, demonstrating high encapsulation efficiency, drug loading, and nasal-brain targeting potential, supporting safe and effective AD treatment [75]. Bhatt et al. formulated solid lipid nanoparticles (SLNs) with an average particle size (149.2 ± 3.2 nm) and encapsulation efficiency (61.9 ± 2.2%) [76]. In rat models, these nanoparticles significantly improved behavior and reduced oxidative stress, demonstrating notable therapeutic efficacy in HD.

#### 4.1.5. Ferulic Acid

Ferulic acid (FA), also known as 4-hydroxy-3-methoxycinnamic acid, is a phenylpropanoid phenolic acid derived from *Ferula sinkiangensis* K. M. Shen or *Ferula fukanensis* K. M. Shen [77]. FA is a promising inhibitor of pathological Aβ aggregation, rapidly interacting with Aβ monomers/oligomers in vitro, disrupting early fiber formation, and promoting unstable, amorphous aggregates [78]. FA also exhibits potent antioxidant activity, scavenges free radicals, and suppresses reactive oxygen species (ROS) generation. It modulates critical signaling pathways (ERK, PI3K/AKT) involved in oxidative stress regulation, mitigating oxidative stress-mediated neuropathology in AD [79]. However, clinical application of FA is limited due to low bioavailability, limited water solubility (only soluble in hot water), physicochemical instability, and poor lipophilic barrier penetration. Saini et al. developed chitosan-coated SLNs encapsulating FA (51.2% encapsulation efficiency) for intranasal delivery [80]. Chitosan enhanced mucosal adhesion, biocompatibility, and reversibly opened nasal epithelial tight junctions. SLNs increased barrier permeability and drug stability, improving nasal mucosal adhesion, penetration, drug release (72%), and FA’s therapeutic efficacy against AD. Botti et al. synthesized a carboxyl-methylated FA dimer prodrug to optimize loading onto nasal solid lipid particles [81]. This prodrug exhibited stable, sustained FA release, enhanced antioxidant and anti-inflammatory activities, and prolonged cerebrospinal fluid retention (120 min). Compared to monomeric FA, chitosan-coated FA provided 7-fold higher brain targeting, fully leveraging antioxidant and anti-inflammatory effects.

**Table 2:** Phenolic and phenolic acid natural products for the nasal treatment of NDs.

Natural Products	Diseases	Experimental Animal Model/Cell Model	Drug Delivery Systems	Formulation Optimization Effects	Pharmacological Effects	Reference
Curcumin	AD	Intraperitoneal injection of scopolamine induced AD in model rats	Curcumin α- and β-d-glucoside isomers		Glutathione and acetylcholine levels↑; Lipid peroxidation and protein carbonyl levels ↓	[55]
		Mimicking mucin in amyloid peptide plaques	Curcumin/Demethoxycurcumin Polymer Nanoparticles	Good capture and released stability	Curcumin had a better affinity for pathological products	[56]
	PD	in vitro: α-syn-mCherry overexpression in SH-SY5Y cells; in vivo: MPTP-induced PD rats	Exosome self-directed nanoparticles	Penetrates nasal mucosal barrier and cell membrane barrier ↑; Brain targeting ↑	Aggregation of α-syn ↓; Neuronal synapse growth, remodelling and functional recovery ↑	[57]
Resveratrol	AD	in vitro: goat nasal mucosa; in vivo: rat	Coconut oil resveratrol nanoemulsion	in vitro drug release: 92.66 ± 3.45%; in vitro permeability of nasal mucosa: 88.54 ± 2.67%; AUC _brain_: 30,049.63 ± 1652.8; C_max brain_: 5762.30 ± 316.9 ng/mL		[62]
		AD model mice were induced by intracerebroventricular injection of lipopolysaccharide	Chitosan-coated RES and superparamagnetic iron oxide nanoparticle-loaded vesicles	Brain targeting ↑; Slow-release performance ↑, Extend resveratrol release for 24 h; Burst effect slightly decreased to 9.6%	Memory and cognitive function ↑; The levels of pro-inflammatory markers, NF-κB and p38 protein ↓; Improved the brain parenchyma and limited the activation of astrocytes in brain tissue.	[63]
		Intracerebroventricular injection of streptozotocin induced an AD model in Swiss female mice	RES nasal in situ gelling system	Blocking property ↑	Spatial memory level ↑; IL-1β, Neuroinflammation ↓	[64]
		APP-, tau-, and α-synuclein-transgenic mice	Combination drugs of rifampicin and resveratrol		Concentrations of brain-derived neurotrophic factor (BDNF) and its precursor, pro-BDNF, in hippocampus ↑; Rifampicin-induced liver injury ↓; Accumulation of amyloid oligomers ↓	[65]
		in vitro: sheep nasal mucosa; in vivo: rat	Hyaluronic acid-adhesive lipid NE encapsulating RSV and Curcumin	Nasal retention time ↑; Mucociliary clearance ↓; Stability of NE↑; AUC_brain_ increased 7 times		[66]
Paeonol	NDs	Goat nasal mucosa; RPMI2650 cells	Paeonol SLNs in situ gel	Nasal mucosa adhesion ↑; Nasal mucosa irritation ↓; Nasal-brain transport efficiency ↑; Sustained release capacity ↑ (72 h sustained release drug 64.89% ± 3.14%)		[70]
Rosmarinic acid	AD	hCMEC/D3 cells	NLC carrying Rosmarinic acid	Drug sustained release time ↑; Transmembrane permeability in vitro ↑	Antioxidant activity ↑	[75]
	HD	HD model rats induced by 3-nitropropionic acid	Rosmarinic acid SLNs	AUC_brain_ ↑; Duration of drug release ↑ (drug concentration is maintained to 14 h)	Lipid peroxidation, nitrite concentration ↓; antioxidant enzyme activity ↑	[76]
Ferulic acid	AD	Goat nasal mucosa; AD model rats were induced by streptozotocin injection	Ferulic acid-loaded chitosan-coated SLNs	In vitro mucosal adhesion and permeability ↑; the drug release rate in vitro increased to 72%; Brain drug levels ↑	Improvement of cognitive ability ↑	[80]
	NDs	in vitro: PC12 cells; in vivo: rat	Dimeric ferulic acid conjugate as prodrug loaded ferulic acid solid lipid microparticles	The drug retention time in cerebrospinal fluid was prolonged and could be detected after 120 min; Bioavaolability ↑; Drug entering brain time was shortened, reaching C_max_ in 20 min; The brain targeting was 7 times higher than monomer		[81]

↑ indicates increase; ↓ denotes decrease.

### 4.2. Glucosides

**Figure 3:**
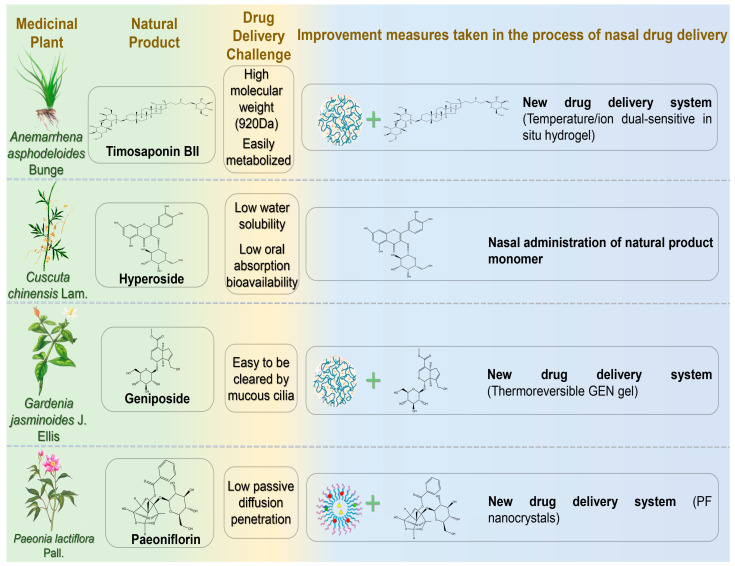
The challenges associated with the treatment of neurodegenerative diseases via nasal administration of glucoside natural products and the schematic diagram illustrating potential solutions. Abbreviations: GEN, geniposide; PF, Paeoniflorin.

Glucosides, an important class of bioactive natural products in traditional Chinese medicine (TCM), exhibit significant neuroprotective properties [82]. Contemporary pharmacological studies have further confirmed their efficacy against NDs. Their therapeutic mechanisms include antioxidation, neurotransmitter regulation, anti-apoptosis, anti-inflammation, attenuation of Ca^2+^ influx, neurotrophic factor regulation, inhibition of tau phosphorylation, and neuronal regeneration. Below, glycoside compounds administered nasally for NDs treatment are summarized (Figure 3) (Table 3).

#### 4.2.1. Timosaponin BII

Timosaponin BII (TBII) is an active compound extracted from the dried rhizome of *Anemarrhena asphodeloides* Bunge, exhibiting pharmacological activities such as improving learning and memory, anti-dementia, antidepressant, anti-inflammatory, anti-tumor, and hypoglycemic effects [83]. TBII significantly suppresses acetylcholinesterase (AChE) activity in mouse cerebral cortex and hippocampus, upregulates acetylcholine M1 receptors, and downregulates inflammatory factors (iNOS, TNF-α, and IL-1β) [84]. Furthermore, it increases GSH-Px and SOD activities and decreases MDA levels, thereby mitigating oxidative stress [85]. These findings suggest therapeutic benefits of TBII for cognitive and behavioral disorders. However, its high molecular weight (920 Da), numerous hydrogen bond donors/acceptors, and susceptibility to metabolism limit oral absorption and complicate in vivo administration [86]. Chen et al. developed a temperature/ion dual-sensitive TBII nasal hydrogel formulation [87]. This formulation achieved rapid gelation, prolonged nasal retention, and inhibited inducible nitric oxide synthase expression. In lipopolysaccharide-induced AD model rats, the hydrogel effectively reduced pro-inflammatory mediators (TNF-α and IL-1β) and inflammatory damage. Following IN, TBII accumulated rapidly in the brain (within 5 min, peaking at 90 min), demonstrating superior brain targeting compared to oral delivery.

#### 4.2.2. Hyperoside

Hyperoside (HYP), a flavonol glycoside extracted from *Cuscuta chinensis* Lam., exhibits antifibrotic, anti-inflammatory, antiviral, and antidepressant properties. Research has indicated HYP may effectively prevent and treat AD by enhancing synaptic function and memory [88]. HYP improved cognitive functions in APP/PS1 transgenic mice by downregulating BACE1 and GSK3β, thereby reducing amyloid plaque deposition and tau phosphorylation. Additionally, HYP inhibited microglia and astrocyte activation, mitigating neuroinflammation and oxidative stress [89]. However, poor water solubility, limited dissolution, and low oral bioavailability (approximately 10%) restrict its pharmacological potential [90]. Song et al. evaluated the pharmacokinetics of intranasal HYP in rat brains, using IV administration as a reference [91]. IN resulted in higher brain absorption and prolonged retention of HYP, with an elimination half-life (70.06 ± 5.04 h) seven times longer than IV (10.4 ± 1.63 h). Compared to IV administration, intranasal delivery provided simpler operation and easier BBB penetration.

#### 4.2.3. Geniposide

Geniposide (GEN), a cyclic enol ether terpene glycoside isolated from *Gardenia jasminoides* J. Ellis (Rubiaceae), possesses diverse medicinal properties, including neuroprotective, antidiabetic, anti-inflammatory, antidepressant, and antioxidant effects [92]. Recent studies have proposed GEN as a potential therapeutic agent for AD due to its pharmacological actions of inhibiting oxidative stress and abnormal Aβ deposition, reducing apoptosis, and protecting neurons [93,94]. Wang et al. developed a thermoreversible GEN gel to enhance bioavailability by extending nasal residence time [95]. The optimized formulation achieved drug content between 97% and 101%, with an average GEN release rate of 99.4% over 6 h. This gel formulation holds promising clinical potential for AD treatment.

#### 4.2.4. Paeoniflorin

Paeoniflorin (PF) is an active glycoside extracted from the dried roots of *Paeonia lactiflora* Pall. or *Paeonia veitchii* Lynch. Recent studies demonstrated that PF activates the HSF1-NRF1 pathway, enhancing antioxidant and anti-inflammatory defenses, reducing oxidative stress and neuroinflammation in PD models, thus preserving dopamine neurons and alleviating pathological symptoms [96]. However, PF’s neuroprotective efficacy is restricted by low passive diffusion, poor bioavailability, and the BBB [97]. Wu et al. developed PF nanocrystals for intranasal brain-targeted PD treatment [98]. The nanocrystals significantly enhanced mucosal barrier penetration, achieving cumulative permeability (87.14% ± 5.34% over 24 h), superior to the free form. Following IN, brain maximum concentration (C_max_) reached 108.47 ± 9.51 ng/g, significantly higher than IV injection (47.19 ± 5.69 ng/g).

**Table 3:** Glucoside natural products for the nasal treatment of NDs.

Natural Products	Diseases	Experimental Animal Model/Cell Model	Drug Delivery Systems	Formulation Optimization Effects	Pharmacological Effects	Reference
Timosaponin BII	AD	in vitro: Sheep nasal septum mucosa; Lipopolysaccharide damaged PC12 cells; in vivo: AD model mice were induced by injection of lipopolysaccharide	Temperature/ion sensitive in situ hydrogel of Timosaponin BII	Brain targeting ↑ (concentrated in brain at 5 min after IN, brain fluorescence intensity was the highest at 90 min after administration); Nasal mucosal retention time ↑	iNOS ↓; Inflammatory cytokines TNF-α and IL-1β ↓	[87]
Hyperin		APP/PSEN1 double transgenic AD mouse model	Hyperin	The drug elimination half-life was 7 times longer than that of intravenous administration (iv); Bioavaolability ↑	Spatial learning and memory ability ↑; Aβ plaques and GFAP levels in cortex and hippocampus ↓	[91]
Geniposide		Goat nasal mucosa	Geniposide thermoreversible in situ gel	Nasal mucosa adhesion ↑; Nasal residence time ↑; Thermal reversibility ↑; The prescription drug content can reach 97 –101%; Gel strength 25–50 s		[95]
Paeoniflorin	PD	in vitro: porcine nasal mucosa; calu-3 cells; SH-SY5Y cells; in vivo: SD rats	Paeoniflorin nanocrystal preparation	AUC_brain_ ↑(AUC_brain 0–8h(IN)_(230.86 ± 18.56 ng.h/g) > AUC_brain 0–8h(iv)_(94.11 ± 10.37 ng.h/g)); Nasal permeability ↑; The cumulative release rate of the drug in 24 h: 87.14% ± 5.34%; Brain targeting ↑ (The C_max IN_ (108.47 ± 9.51 ng/g) was significantly higher than that after IV (47.19 ± 5.69 ng/g))	Neuroprotective effects ↑	[98]

↑ indicates increase; ↓ denotes decrease.

### 4.3. Flavonoids

**Figure 4:**
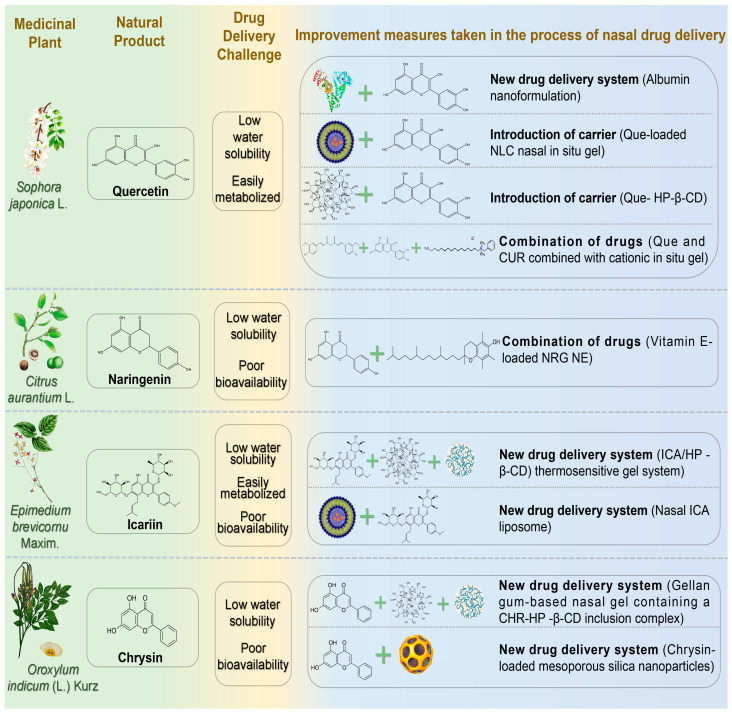
The challenges associated with the treatment of neurodegenerative diseases via nasal administration of flavonoid natural products and the schematic diagram illustrating potential solutions. Abbreviations: Que, Quercetin; HP-β-CD, hydroxypropyl-β-cyclodextrin; NRG, Naringenin; ICA, Icariin; CHR, Chrysin.

Flavonoids are important compounds in developing novel NDs therapeutics, exhibiting neuroprotective effects through antioxidant properties, enhancement of cholinergic neuron function, inhibition of neuronal apoptosis, and modulation of neurotrophic and regenerative pathways (Figure 4) (Table 4) [99].

#### 4.3.1. Quercetin

Quercetin (Que) is a flavonol widely present in natural plants with diverse biological activities, including antibacterial, anti-inflammatory, antioxidant, and anti-proliferative effects. Que is lipophilic and readily crosses the BBB, making it a promising candidate for treating neurodevelopmental disorders [100]. Sabogal-Guáqueta et al. demonstrated Que’s efficacy in reducing amyloidosis and glial proliferation in aged transgenic AD mice, thereby preserving cognitive and emotional functions [101]. However, Que’s oral bioavailability is limited by poor water solubility and peripheral metabolism, restricting its antioxidant and neuroprotective potential [102]. Dou et al. incorporated Que into human serum albumin, developing a natural plant antioxidant nanoformulation for advanced AD [103]. Their formulation exhibited sustained release, nearly complete within 24 h. It reduced weight loss, improved survival rates, and significantly reduced oxidative stress, Aβ aggregation, and synaptic damage, reversing severe cognitive impairment in model rats. Sonawane and Pokharkar developed Que-loaded nanostructured lipid carrier (Que-NLC) nasal gels, achieving significant therapeutic concentrations (C_max_ = 183.41 ± 11.76 ng/mL in CNS) [104]. This formulation exhibited targeting efficiency (117.47%) and targeting potential (88.9%), surpassing the anti-AD activity of donepezil (positive control). Manta et al. constructed a Que-hydroxypropyl-β-cyclodextrin (HP-β-CD) complex, significantly enhancing Que’s water solubility (50-fold increase) [105]. The complex exhibited favorable permeability across rabbit nasal mucosa. Vaz et al. developed a nasal nanoformulation combining natural antioxidants Que and CUR for anti-NDs treatment [106]. The optimized formulation incorporated cationic agents for enhanced mucosal adhesion and permeability and in situ gelling agents to prolong nasal retention. Safety and efficacy assessments showed significantly improved drug release and enhanced anti-NDs performance compared to free Que and CUR.

#### 4.3.2. Naringenin

Naringenin (NRG) is a natural flavonoid aglycone widely found in herbal medicines such as Fructus Aurantii Immaturus, Dendrobium officinale, and Fructus Aurantii. Recently, the neuroprotective effects of NRG have attracted increasing attention, particularly for PD treatment [107]. NRG exerts neuroprotective actions and promotes neural regeneration by activating the Nrf2 pathway. This activation stimulates the secretion of neurotrophic factors, including BDNF and GDNF, in dopaminergic neurons and functions as an astrocyte-derived neurotrophic factor [108]. However, poor bioavailability and water insolubility constrain the clinical application of NRG. Gaba et al. developed a vitamin E-loaded NRG nanoemulsion (particle size 38.70 ± 3.11 nm; viscosity 19.67 ± 0.25 Pas) for intranasal brain delivery in PD [109]. Vitamin E served as an antioxidant, synergizing with NRG. Compared to free NRG, this nanoemulsion enhanced nasal permeability and release nearly threefold and significantly increased brain uptake. After 30 min, brain NRG concentration reached 394.33 ± 80.83 ng/mL, approximately twice the intravenous administration, significantly enhancing brain bioavailability (334.20 ± 8.91%). This study provided effective evidence supporting NRG’s potential in treating PD-related symptoms.

#### 4.3.3. Icariin

Icariin (ICA) is a major flavonoid found in *Epimedium brevicornu* Maxim. Modern pharmacological studies demonstrate therapeutic potential for ICA in treating NDs, cardiovascular diseases, osteoporosis, and tumors [110]. Chen et al. demonstrated ICA’s neuroprotective effects in MPTP-induced PD mouse models by inhibiting apoptosis and enhancing neuronal survival through activation of PI3K/Akt and MEK/ERK pathways [111]. Although ICA can cross the BBB, its poor aqueous solubility, low oral bioavailability, and rapid clearance severely restrict research and clinical application [112]. Therefore, novel drug delivery systems are essential. Wang developed an ICA/hydroxypropyl-β-cyclodextrin (HP-β-CD) delivery system, significantly enhancing ICA’s solubility, bioavailability, and controlled release [113]. IN of ICA-loaded thermosensitive gel achieved nearly complete drug release (98.64 ± 1.08%) within 24 h, effectively treating PD. Cheng et al. developed nasal ICA liposomes to enhance brain targeting and bioavailability [114]. Behavioral tests in PD rats indicated significant improvements, including increased rotational behavior, enhanced bilateral forelimb function, inhibited caspase-3 activation in substantia nigra, and restored motor function and neurotransmitter balance.

#### 4.3.4. Chrysin

Chrysin (CHR), a natural flavonoid derived from *Oroxylum indicum* (L.) Kurz, demonstrates diverse pharmacological properties, including anti-inflammatory, antioxidant, and cardiocerebrovascular protective effects [115]. CHR exhibits neuroprotection against PD via multi-target pathways, primarily mediated by antioxidant and anti-apoptotic actions. Specifically, CHR activates the Nrf2/ARE signaling pathway, upregulating antioxidant enzymes (SOD, GSH, and HO-1) and reducing oxidative stress-mediated dopaminergic neurodegeneration [116]. Concurrently, it modulates the Bcl-2/Bax balance to inhibit apoptosis and potentially disrupt α-synuclein aggregation [117]. However, CHR’s poor solubility and bioavailability limit its clinical application [118]. Advanced drug delivery systems or optimized delivery strategies may enhance therapeutic efficacy. Khushboo and Anuj formulated a gellan gum-based nasal gel containing a CHR-hydroxypropyl-β-cyclodextrin inclusion complex, exhibiting sustained release, high permeability, and neuroprotective properties [119]. To overcome poor aqueous solubility, Shital et al. developed mesoporous silica nanoparticle (MSN) carriers loaded with CHR for targeted intranasal brain delivery [120]. The CHR-loaded MSNs represented a non-invasive therapeutic approach for NDs, combining high drug payload, pH-responsive release kinetics, and olfactory-mediated delivery. This formulation achieved sustained release, reduced systemic toxicity, and synergistic antioxidant effects, enhancing targeted neuroprotection and therapeutic indices.

**Table 4:** Flavonoid natural products for the nasal treatment of NDs.

Natural Products	Diseases	Experimental Animal Model/Cell Model	Drug Delivery Systems	Formulation Optimization Effects	Pharmacological Effects	Reference
Quercetin	AD	in vitro: H_2_O_2_-induced oxidative damage in PC12 cells; in vivo: APP/PS1 AD model mice	Que-coated human serum albumin NPs	Sustained release capacity ↑ (24 h can reach 99% release rate)	Oxidative stress ↓; Aβ aggregation ↓; Neuronal apoptosis ↓; synaptic damage in brain ↓	[103]
		in vitro: Goat nasal mucosa; in vivo: Trimethyltin-induced NDs rats	Quercetin NLC in situ gel	Brain targeting ↑ (drug targeting efficiency: 117.47%); Brain drug accumulation ↑ (C_max_: 183.41 ± 11.76 ng/mL); In vitro drug release ↑ (in vitro drug release rate: 83.74 ± 1.40%); Drug solubility ↑; Nasal mucosal retention time ↑		[104]
		Rabbit nasal mucosa	β-cyclodextrin derivative Que inclusion complex	The solubility of Que was 50 times higher than monomer; Nasal mucosal barrier permeability ↑		[105]
	NDs	RPMI2650 cells	Quercetin NE	Drug loading ↑; Gel-modified NE particle size increased to 244 nm, the entrapment efficiencyvalues were higher than 99%; Stability ↑	Antioxidant activity ↑	[106]
Naringenin	PD	in vitro: Goat nasal mucosa; in vivo: 6-OHDA-induced PD rat model	Vitamin E-loaded naringenin NE	The drug release rate was 3 times higher than monomer; The permeability of nasal mucosa increased by 3 times; C_brain_↑ (394.33 ± 80.83 ng/mL); Brain bioavailability: 334.20 ± 8.91%	Antioxidant activity ↑	[109]
Icariin	PD	Paraquat-induced PD mouse model	Icariin/hydroxypropyl-β-cyclodextrin inclusion complex controlled-release thermosensitive gel	Duration of drug release ↑ (the cumulative release of 24 h reached 98.64 ± 1.08%); Drug stability, solubility ↑; Bioavailability, brain targeting ↑	Antioxidant activity ↑	[113]
		6-OHDA-induced PD rat model	Icariin liposome		Necrosis and apoptosis of dopaminergic neurons in substantia nigra ↓	[114]
Chrysin	PD	Sheep nasal mucosa; SH-SY5Y cell model induced by H_2_O_2_	Gellan gum nasal gel loaded with chrysin/HP-β-CD inclusion complex	Sustained release capacity ↑ (12 h can reach 78.27 ± 3.81% accumulated release rate); The permeability of nasal mucosa ↑ (accumulative permeability reached 78.73 ± 0.37%); Stability ↑ (The drug content remained 97.49 ± 0.34% within 3 months)	Cell viability ↑ (*p* < 0.05); Superoxide dismutase (SOD) enzymatic activity ↑	[119]
	NDs	OBGF400cells	mesoporous silica nanoparticles based delivery system	Drug loading ↑ (11.49 ± 1.19% *w*/*w*); PH-responsive sustained release; Stability ↑	Regulating oxidative stress pathway; cytotoxicity ↓	[120]

↑ indicates increase; ↓ denotes decrease.

### 4.4. Alkaloid

**Figure 5:**
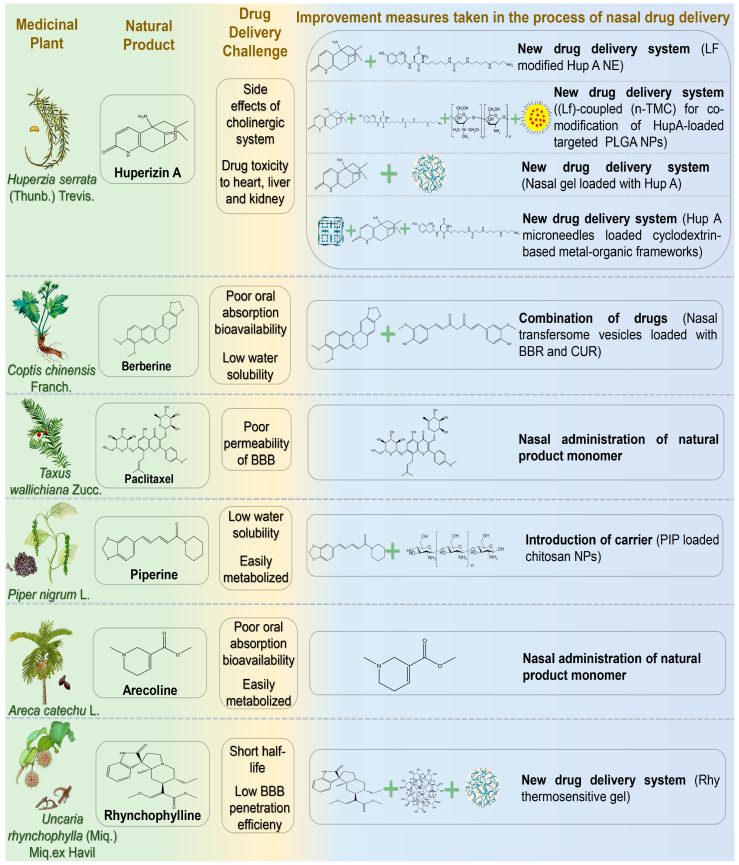
The challenges associated with the treatment of neurodegenerative diseases via nasal administration of alkaloid natural products and the schematic diagram illustrating potential solutions. Abbreviations: LF, lactoferrin; Hup, Huperzine A; n-TMC, n-trimethyl chitosan; PLGA, polylactic acid-glycolic acid; BBR, berberine; PIP, Piperine; Rhy, Rhynchophylline.

Alkaloids are common nitrogen-containing organic compounds found widely in plants. Due to their nitrogenous structures, alkaloids are considered promising therapeutic candidates for NDs [121]. Nasal administration of natural product-derived alkaloids has recently become a research focus (Figure 5) (Table 5). Relevant research progress is summarised as follows.

#### 4.4.1. Huperizin A

Huperizin A (HupA) is an unsaturated sesquiterpene alkaloid extracted from *Huperzia serrata* (Thunb.) Trevis. As an efficient and reversible AChE inhibitor, HupA has been widely employed in the treatment of cognitive dysfunction, AD, and other NDs [122]. Additionally, HupA exhibits potential anti-aging, anti-apoptotic, and neuroprotective effects against amyloid-induced neuronal oxidative damage and mitochondrial dysfunction. Possible mechanisms include inhibition of the NF-κB pathway, upregulation of nerve growth factor, and modulation of N-methyl-D-aspartate receptors [123]. However, oral administration of HupA may interfere with the cholinergic system due to peripheral metabolism. As a lipophilic weak alkaloid, HupA exhibits favorable permeability and can reach the CNS via the nasal passage. Jiang et al. developed a lactoferrin-modified HupA nanoemulsion for IN [124]. Lf-modified HupA significantly prolonged the drug action time in the brain, enabling sustained release (24 h release rate of 85%). Nanoemulsion (NE) delivery has been shown to inhibit efflux pumps and enhance drug absorption. The drug targeting index (DTI) in the brain (3.2 ± 0.75) indicated effective targeting, reducing drug toxicity to the heart, liver, and kidneys. Compared with intragastric administration, intranasal delivery of HupA provided faster absorption, more effective drug distribution, and sustained release to the brain. Meng et al. developed lactoferrin (Lf)-coupled N-trimethyl chitosan (n-TMC)-co-modified HupA-loaded polylactic acid-glycolic acid (PLGA) nanoparticles (NPs) [125]. This delivery system combined the advantages of Lf in enhancing nasal-brain transmission, n-TMC with strong adhesion and solubility, and PLGA NPs with high targeting and low toxicity. Lf-TMC NPs continuously released HupA for 48 h after IN and promoted brain distribution. The DTIs of Lf-TMC NPs in the olfactory bulb, brain (excluding hippocampus), cerebellum, and hippocampus of model mice were 2.0, 1.6, 1.9, and 1.9, respectively. Wang et al. prepared a nasal gel containing HupA to explore its novel mechanism in reducing AD-associated brain amyloidosis [126]. After administering HupA to model mice, the researchers evaluated its safety. They found no significant structural changes in the olfactory bulb, neurogenesis in the subventricular zone (SVZ), or BBB permeability. These results indicated no apparent brain toxicity from intranasal HupA. Regarding the mechanism, the study revealed a novel perspective: HupA upregulated β-catenin expression both in vivo and in vitro, suggesting that its neuroprotective effect may involve regulation of the Wnt signaling pathway in AD. Ruan et al. developed an innovative transnasal intracerebral delivery system using microneedles loaded with a cyclodextrin-based metal-organic framework as a HupA carrier [127]. The nanocarrier was modified with LF and incorporated into a dissolving microneedle patch based on hyaluronic acid and gelatin. The microneedle served as an active drug delivery device, enabling precise drug delivery by minimally invasive penetration of physiological barriers. This novel delivery system demonstrated enhanced physical stability and favorable biocompatibility. Compared with free drug delivery, microneedle-assisted nasal penetration increased 1.7-fold, achieving efficient nasal-brain targeted HupA delivery. This significantly improved memory impairment in rats and may provide a novel nasal treatment option for NDs.

#### 4.4.2. Berberine

Berberine (BBR) is an isoquinoline alkaloid extracted from *Coptis chinensis* Franch. that exhibits various pharmacological effects, including antibacterial, anti-diabetic, anti-cancer, anti-inflammatory, antioxidant, and anti-tumor properties [128]. BBR can alleviate cognitive decline in AD patients by inhibiting extracellular amyloid plaque deposition and intracellular neurofibrillary tangles, as well as by enhancing autophagic activity [129]. However, oral administration of BBR has low bioavailability due to poor solubility, extensive intestinal first-pass metabolism mediated by cytochrome P450 enzymes and β-glycoprotein efflux transporters, and hepatic first-pass metabolism [130]. Additionally, intramuscular and intravenous administration of BBR may induce allergic reactions. Therefore, the development of new drug delivery systems to improve BBR bioavailability has attracted considerable attention from drug developers [131]. Mishra et al. prepared nasal transfersome vesicles loaded with BBR and CUR (BBR-CUR-trans) [132]. Targeted dual drug-loaded nanoparticles were developed to prevent off-target effects associated with single-molecule drug delivery. In vitro studies and safety evaluations demonstrated that BBR-CUR-trans provided sustained drug release for 6 h and had a lower hemolysis rate compared with pure BBR and CUR. After IN, BBR-CUR-trans exhibited effective brain targeting, and the average brain residence time was six times longer than that of the drug-only group. Furthermore, behavioral studies showed that BBR-CUR-trans significantly reduced pro-inflammatory cytokine production in the cortex and hippocampus. The inhibition of AChE was also significantly more effective than pure drugs, suggesting a synergistic effect of BBR and CUR. Transfersome vesicles effectively transported drugs across the BBB and exhibited enhanced transport capacity within the brain.

#### 4.4.3. Paclitaxel

Paclitaxel (PTX) is a tetracyclic diterpene anti-tumor compound isolated and purified from Taxus species in the 1990s, possessing microtubule-polymerizing and stabilizing properties [133]. As a microtubule-stabilizing agent, PTX exerts therapeutic effects in AD and related tauopathies by modulating microtubule dynamics, reducing tau hyperphosphorylation, and mitigating tau-related neurodegeneration [134]. However, many microtubule stabilizers, including paclitaxel, reduce BBB permeability. Therefore, oral administration limits its therapeutic efficacy in CNS disorders. Studies exploring paclitaxel as a nasal preparation for anti-tumor purposes have gradually emerged and shown promising results. Cross et al. investigated whether intranasal paclitaxel could alter disease progression in animal models of AD [135]. Following administration, PTX prevented axonal transport impairment and reduced phosphorylated tau in neurons. Moreover, paclitaxel-treated AD mice demonstrated significantly improved cognitive function in the Morris water maze test.

#### 4.4.4. Piperine

Piperine (PIP) is an amide alkaloid isolated from *Piper nigrum* L., exhibiting pharmacological effects in AD prevention and treatment through anti-inflammatory and antioxidant activities, inhibition of AChE, and suppression of Aβ aggregation [136,137]. However, the hydrophobicity of PIP and cytochrome P450-mediated first-pass metabolism restrict its full entry into the brain when administered orally [138]. Elnaggar et al. prepared PIP-loaded chitosan NPs for nasal administration [139]. Chitosan NPs offer advantages such as biocompatibility, biodegradability, non-toxicity, and low cost, significantly enhancing drug stability and absorption. In vitro studies demonstrated a cumulative release rate of 92% for PIP after 24 h. Behavioral tests confirmed that these NPs provided effective brain-targeted therapy, improving cognitive function comparable to donepezil.

#### 4.4.5. Arecoline

Arecoline (ARE) is the main active ingredient from the palm plant *Areca catechu* L., which has a long-standing tradition of medicinal use in China. Recently, ARE has been found to effectively alleviate and repair nerve injury [140]. As an M receptor agonist, ARE stimulates the M receptor, compensating for the progressive decline in acetylcholine levels in the brains of AD patients, thus enhancing cognitive function [141]. Due to its ability to cross the BBB, ARE is considered a promising candidate for treating CNS diseases. However, low oral bioavailability and rapid systemic clearance significantly hinder its pharmacodynamic effects [142]. Hussain and Mollica verified the efficacy of nasal ARE in AD, reporting rapid absorption (T_absorption_ = 3 ± 1.6 min) and a swift plasma concentration peak [143].

#### 4.4.6. Rhynchophylline

Rhynchophylline (Rhy) is an indole alkaloid extracted from the stems of *Uncaria rhynchophylla* (Miq.) Miq.ex Havil, possessing therapeutic effects including antihypertensive, antithrombotic, antiasthmatic, and anticancer properties [144]. Recent research has demonstrated its neuroprotective effects in PD by scavenging oxygen free radicals, inhibiting inflammatory responses, and improving antioxidant capacity [145]. Lin et al. developed a nasal Rhy thermosensitive gel composed of poloxamer 407, poloxamer 188, PEG-6000, and HP-β-CD in specific ratios [146]. The gel exhibited temperature-responsive properties, facilitating phase transition and adherence to lesion sites for prolonged drug release.

**Table 5:** Alkaloid natural products for the nasal treatment of NDs.

Natural Products	Diseases	Experimental Animal Model/Cell Model	Drug Delivery Systems	Formulation Optimization Effects	Pharmacological Effects	Reference
Huperzine A	AD	in vitro: Human cerebral microvascular endothelial cells/D3; in vivo: Wistar rats	LF modified Hup A NE	Drug solubility ↑; Stability ↑; Sustained release capacity ↑ (24 h release rate of 85%); Brain targeting ↑ (Drug targeting indexes (DTI) in brain (3.2 ± 0.75) showed effective targeting)		[124]
		in vitro: Human bronchial epithelial cell line 16HBE cells and SH-SY5Y cells; in vivo: Kunming mice	LF-coupled n-TMC was used to co-modify targeted PLGA NPs loaded with Hup A	Sustained release ability ↑ (48 h release rate: 74.5% ± 4.5%); Adhesion ↑ (the adhesion of NPs modified by TMC increased by nearly three times); Brain targeting ↑; Cytotoxicity ↓		[125]
		in vitro: SH-SY5Y cells in vivo: APP/PS1 double transgenic mice	Hup A nasal gel		Antioxidant enzyme activity ↑; Inhibition of acetylcholinesterase activity ↑; Aβ aggregation ↓	[126]
		in vitro: 16HBE cells, PC12 cells; Pig nasal mucosa; in vivo: AD model rats induced by hyoscyamine and d-galactose	Cyclodextrin-based metal-organic framework-mediated Hup A nanomicroneedles	Administration time ↓; Physical stability ↑; Nasal mucosal penetration ability ↑ (the amount of nasal penetration of the drug was 1.7 times higher than free); Brain uptake rate ↑; Brain targeting ↑	Neuronal cell injury ↓; Spatial memory improvement ability ↑	[127]
Berberine	AD	AD mice were induced by intranasal administration of scopolamine	Nasal transfersome vesicles loaded with berberine and curcumin	Sustained release ability ↑ (60 h release saturation); Hemolytic toxicity ↓; The average residence time in brain was 7 times than pure drug group	Inhibition of Acetylcholinesterase ↑; Spatial memory improvement ability ↑; Antioxidant enzyme activity ↑	[132]
Paclitaxel	AD	in vitro: Primary hippocampal neurons of E18 embryonic rats; in vivo: 3XTg-AD mice;	Paclitaxel		AD model mice cognitive impairment ↓; Hyperphosphorylation of tau protein and abnormal proliferation of glial cells ↓; Axonal transport in body ↑	[135]
Piperine	AD	Intraventricular injection of colchicine-induced SD model rats	PIP-loaded chitosan NPs	Brain targeting ↑; Nasal mucosa irritation ↓; The dose was reduced by 20 times compared with oral administration ↓; Sustained release time ↑ (24 h cumulative release rate of 92%)	Acetylcholinesterase inhibition and antioxidant effect ↑	[139]
Arecoline	AD	Male Lewis rats	Arecoline	Drug absorption rate ↑ (t = 3 ± 1.6 min); Drug elimination half-life ↑		[143]
Rhynchophylline	PD	in vitro: SH-SY5Y cells; in vivo: MPTP+ induced PD model mice	Rhy thermosensitive gel	Nasal mucosal permeability ↑; Nasal mucosa adhesion ↑; Slow-release performance ↑; Bioavailability ↑; Brain targeting ↑ (DTI is 2.1 times higher than oral administration)	Abnormal expression of oxidative stress factors, neurons positive damage, and dopamine in substantia nigra ↓	[146]

↑ indicates increase; ↓ denotes decrease.

### 4.5. Terpenoids

**Figure 6:**
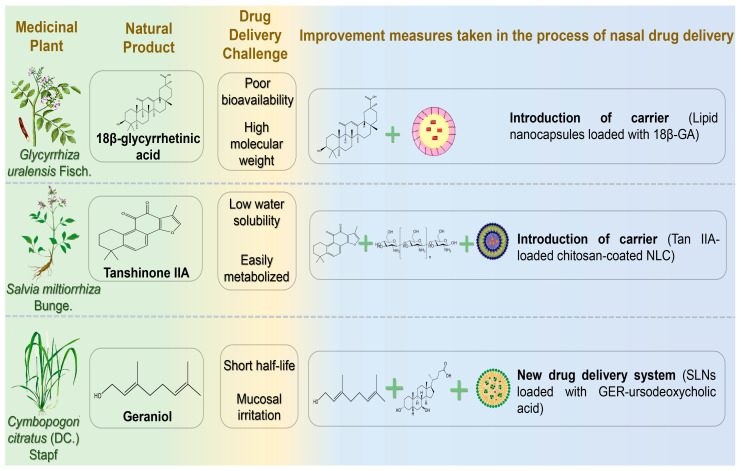
The challenges associated with the treatment of neurodegenerative diseases via nasal administration of terpenoid natural products and the schematic diagram illustrating potential solutions. Abbreviations: 18β-GA,18β-glycyrrhetinic acid; Tan IIA, tanshinone IIA; GER, geraniol.

Terpenoids, structurally diverse secondary metabolites, are widely distributed in nature and exhibit a broad spectrum of biological activities. Over recent decades, research into their neuroprotective effects has significantly increased due to their strong anti-inflammatory and neuroprotective properties (Figure 6) (Table 6) [147].

#### 4.5.1. 18β-Glycyrrhetinic Acid

18β-glycyrrhetinic acid (18β-GA) is a triterpenoid compound extracted from the dried roots and rhizomes of the leguminous plants *Glycyrrhiza uralensis* Fisch., *Glycyrrhiza inflata* Bat., and *Glycyrrhiza glabra* L. 18β-GA exerts neuroprotective effects by suppressing inflammatory factor expression and modulating the PI3K/Akt signaling pathway. Additionally, it mitigates ROS accumulation associated with AD by scavenging hydroxyl and superoxide radicals, subsequently promoting neuronal proliferation [148,149]. However, the clinical application of 18β-GA is limited by high molecular weight, low bioavailability, and poor water solubility. Gad et al. designed lipid nanocapsules (LNCs) encapsulating 18β-GA to enhance nasal delivery, overcoming BBB penetration and first-pass metabolism challenges [150]. Optimized LNCs containing 18β-GA exhibited nanoscale particle size, good stability for 6 months, slow and sustained drug release within 24 h, and high steady-state flux and nasal mucosal permeability within 8 h. Morris water maze tests demonstrated improved spatial memory with this drug delivery system.

#### 4.5.2. Tanshinone IIA

Tanshinone is a predominant diterpenoid quinone found in the roots and rhizomes of *Salvia miltiorrhiza* Bunge, attracting attention for its potential in PD treatment, particularly Tanshinone IIA (Tan IIA) [151]. The neuroprotective effects of Tan IIA in PD are primarily mediated by inhibiting α-synuclein oligomerization and fibrillation, attenuating damage to nigrostriatal dopaminergic neurons, reducing inflammatory responses in glial cells, and mitigating oxidative stress [152]. However, clinical utility is limited by its low water solubility, poor permeability, and short half-life due to first-pass hepatic metabolism. Hassan et al. prepared chitosan-encapsulated nanostructured lipid carriers (NLCs) loaded with Tan IIA using melt emulsification sonication, achieving a high encapsulation efficiency (97%) [153]. Due to the adhesion and permeability properties of chitosan, NLC residence time in the nasal cavity was extended, achieving sustained drug release over 24 h following IN.

#### 4.5.3. Geraniol

Geraniol (GER), a monoterpene alcohol extracted from the leaves of *Cymbopogon citratus* (DC.) Stapf, exhibits multiple bioactivities, including antibacterial, anti-inflammatory, analgesic, anti-asthmatic, and anti-tumor effects [154]. Previous research demonstrated that GER exerts neuroprotective effects in in vitro PD models by reducing oxidative stress-induced neuronal damage through antioxidant mechanisms. Notably, it suppresses inflammatory mediator release, modulates neuroinflammatory pathways, and regulates iron metabolism-associated proteins, thereby preventing pathological iron accumulation in neurons [155]. However, the short half-life of GER in the bloodstream limits its application in long-term treatment via conventional administration routes. Nasal administration allows direct drug absorption from the olfactory region, bypassing the BBB. Unfortunately, GER irritates mucosal tissue, causing olfactory epithelium damage following IN of unmodified GER [156]. Therefore, an effective delivery system is essential to protect the nasal mucosa. De Oliveira et al. synthesized geraniol-ursodeoxycholic acid (GER-UDCA), aiming to combine the anti-inflammatory properties of GER with the mitochondrial rescue effects of UDCA [157]. Due to the poor solubility of both drugs, the researchers embedded them in SLNs to explore nose-to-brain targeting. Following administration, GER-UDCA was detected in rat cerebrospinal fluid but not in blood, suggesting direct nose-to-brain delivery. IN of GER-UDCA-SLNs notably preserved nasal mucosal integrity compared to free GER.

**Table 6:** Terpenoid natural products for the nasal treatment of NDs.

Natural Products	Diseases	Experimental Animal Model/Cell Model	Drug Delivery Systems	Formulation Optimization Effects	Pharmacological Effects	Reference
18β-glycyrrhetinic acid	AD	in vitro: Sheep nasal mucosa; in vivo: scopolamine-induced AD model Wister mice	LNCs coated with 18β-glycyrrhetinic acid	Nasal mucosal permeability ↑; Drug sustained release time 24 h ↑; Brain bioavailability ↑; Nasal brain targeting ↑		[150]
Tanshinone IIA	PD	Rotenone-induced PD rat model	Tan IIA chitosan-coated nano-lipid carrier	Drug solubility ↑; Drug sustained release time 24 h ↑; Nasal mucosal retention time ↑; Brain targeting ↑	Anti-PD activity ↑ (striatal dopamine level ↑; Neuroinflammation and oxidative stress level ↓)	[153]
Geraniol	PD	in vitro: Nasal mucosa of Wistar rats; in vivo: SD rats	Geraniol β-cyclodextrin/hydroxypropyl β-cyclodextrin inclusion complex	Drug solubility ↑ (HP-β-CDs increased the solubilization of GER nearly threefold to 14.73 ± 0.07); Drug stability ↑; Nasal mucosal penetration ability ↑; Brain targeting ↑ (C_(CSF)_GER-HP-β-CD: 1.25 ± 0.03~0.26 ± 0.03 μg/mL; C_(CSF)_GER-β-CD: 119.0 ± 8.6 to 12.6 ± 3.3 μg/mL)		[157]

↑ indicates increase; ↓ denotes decrease.

### 4.6. Others

**Figure 7:**
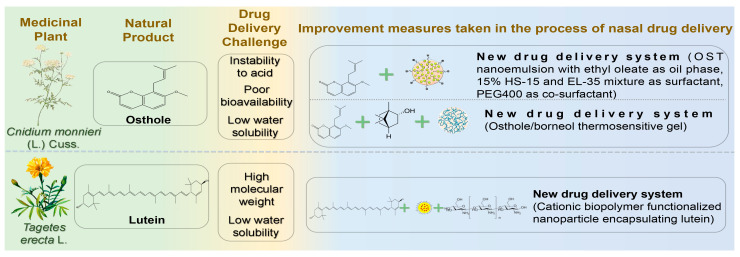
The challenges associated with the treatment of neurodegenerative diseases via nasal administration of other natural products and the schematic diagram illustrating potential solutions. Abbreviations: OST, osthole; HS-15, 15-hydroxystearic acid; EL-35, polyoxyethylene 35 castor oil; PEG400, polyethylene glycol 400.

In addition to the extensively studied compound categories above, coumarin and carotenoid compounds have also been explored recently for nasal delivery in NDs treatment (Figure 7) (Table 7).

#### 4.6.1. Osthole

Osthole (OST) is a natural coumarin extracted from *Cnidium monnieri* (L.) Cuss. It exhibits anti-inflammatory, antioxidative, anti-apoptotic, and anti-tumor activities [158]. Recent studies showed that OST significantly enhanced spatial learning and memory in AD model rats, reduced neuronal apoptosis, and regulated cell cycle progression, thus exerting neuroprotective effects [159]. Additionally, OST ameliorated inflammation in Aβ_1-42-injured BV2 cells by increasing cell viability and reducing inflammatory factor secretion, possibly by downregulating APOE and TREM2 gene and protein expression [160]. However, poor water solubility, low bioavailability, and instability of coumarin components in acidic environments hinder the therapeutic application of OST in nervous system disorders. Difficulty penetrating the BBB through oral administration also restricts OST use in treating encephalopathy [161]. Nanoemulsion (NE) is an optimal drug carrier due to its composition of oil, water, surfactants, and co-surfactants, enhancing the solubility of hydrophobic drugs [162]. Song et al. formulated OST-NE using ethyl oleate as the oil phase, a mixture of 15% HS-15 and EL-35 as surfactants, and PEG400 as a co-surfactant [163]. OST-NE exhibited robust physical stability and significantly enhanced sustained release, achieving 80% OST release within 72 h. Brain retention was notably prolonged, with a 7.08-fold reduction in brain clearance and a 1.73-fold increase in brain-targeting efficiency compared to intravenous administration. Wu et al. prepared an osthole/borneol (OST/BO) gel guided by the “ruler, minister, coordinator and enabler” theory, utilizing BO as the “enabler” to direct OST to the targeted brain area due to its aromatic and orifice-opening properties [164]. The optimized gel demonstrated excellent in vitro release, with OST dissolution exceeding 95% within 300 min. Compared to oral administration, OST concentration in cerebrospinal fluid increased nearly tenfold after IN.

#### 4.6.2. Lutein

Lutein (LT) is a natural carotenoid containing two ionone rings found in *Tagetes erecta* L., an exotic herbal medicine. Recent studies revealed multiple pharmacological effects of LT, including antioxidation, alleviation of visual fatigue, prevention of atherosclerosis, and anti-tumor activity [165]. Furthermore, LT exhibits potent antioxidant properties due to its molecular structure containing long-chain conjugated olefins. Thus, it is considered effective in managing NDs caused by elevated oxidative stress in the brain [166]. Nevertheless, high molecular weight limits LT from crossing the BBB easily. Moreover, its low bioavailability and solubility further restrict its application in CNS diseases. Dhas and Mehta developed cationic biopolymer-functionalized nanoparticles encapsulating LT to combat oxidative stress through IN [167]. Positively charged chitosan strongly adsorbed onto the nasal mucosa, extending residence time and sustaining release. Chitosan was coated onto the surface of PLGA nanoparticles as a shell material, achieving 70.12 ± 1.25% drug release over 96 h. This combination opened tight junctions, enhanced nasal mucosal permeability, and promoted water absorption from the mucus layer, forming a gel matrix to increase contact with absorption sites. This approach provides a potential strategy for effectively treating AD.

**Table 7:** Other natural products for the nasal treatment of NDs.

Natural Products	Diseases	Experimental Animal Model/Cell Model	Drug Delivery Systems	Formulation Optimization Effects	Pharmacological Effects	Reference
Osthole	AD	in vitro: L-glutamic acid-induced excitotoxic SH-SY5Y cells; in vivo: Scopolamine induced AD mice	Osthole NE	Slow-release performance ↑ (72 h release 80% OST); AUC _brain_ ↑; Brain targeting ↑ (1.73 times higher than IV infusion); The brain clearance rate was 7.08 times lower than IV.	Antioxidant enzyme (superoxide dismutase and glutathione) activity ↑	[163]
		in vitro: Nasal mucosa from SD rats and APP/PS1 mice; in vivo: APP/PS1 transgenic mice	OST/Borneol thermosensitive gel	Intercellular space of nasal mucosa ↑; The drug release performance was good (the total dissolution rate of OST exceeded 95% within 300 min.); Brain bioavailability ↑; AUC_brain_ ↑ (C_brain_ is nearly 10 times higher than oral administration)	Clearance of Aβ ↑	[164]
Lutein	AD	in vitro: Goat nasal mucosa; SH-SY5Y cells; U-373MG cells; in vivo: SD rats	Cationic polymer-functionalized NPs carrying LT	Sustained drug release ability ↑ (drug release after 96 h was 70.12 ± 1.25%); Nasal mucosa permeability ↑ (24 h permeability 87.01%); BBB permeability ↑; Brain targeting ↑ (the C_max_ of LT coated with chitosan in brain was 1.7 times higher than uncoated LT)		[167]

↑ indicates increase.

## 5. Conclusions and Future Perspectives

With global population aging, the incidence of NDs, represented by AD, PD, and HD, is increasing annually. The pathogenesis of NDs is complex and diverse, with limited effective clinical treatments available. Consequently, the pursuit of novel therapies for these diseases has become a critical research focus. Compared to Western medicine, TCM offers the advantages of multi-component, multi-target, and multi-effect characteristics. It can simultaneously regulate multiple tissues and molecular targets, reduce toxic side effects, overcome drug resistance, and improve therapeutic efficacy. Clinically, TCM has demonstrated a unique and indispensable role in preventing and treating NDs. Natural products derived from TCM exhibit neuroprotective effects through antioxidative stress, anti-apoptotic activities, mitochondrial function preservation, neuroprotection, and autophagy activation [168]. The neuroprotective mechanisms of saponins include antioxidation, neurotransmitter regulation, anti-apoptosis, anti-inflammation, reduction of Ca^2+^ influx, regulation of neurotrophic factors, inhibition of tau phosphorylation, and promotion of regenerative processes [169]. In intranasal drug delivery systems, saponin derivatives, such as timosaponin BII and geniposide, have shown promising potential. Additionally, ginsenosides have demonstrated multi-target modulatory properties against NDs. For instance, ginsenoside RK1 surpasses donepezil in cognitive enhancement by mitigating Aβ-induced mitochondrial depolarization, reducing oxidative stress via decreased ROS production, and blocking pro-apoptotic neuronal signaling cascades [170]. Meanwhile, ginsenoside Rg1 targets RTP801/α-synuclein to ameliorate autophagy dysfunction, effectively alleviating stress-induced motor and neuropathological impairments in PD model mice [171]. Flavonoids exert neuroprotective effects due to their antioxidant properties and capacity to enhance cholinergic neuronal function, inhibit neuronal apoptosis, and regulate associated neurotrophic and regenerative mechanisms [172]. A representative example, luteolin, ameliorates cognitive decline in 3xTg-AD mice through peroxisome proliferator-activated receptor γ-dependent pathways. Mechanistically, luteolin attenuates Aβ-triggered oxidative stress, restores mitochondrial bioenergetics, and prevents neuronal death, highlighting its multi-target efficacy against AD [173]. Alkaloids, such as matrine and evodiamine, have emerged as promising candidates for NDs intervention. Their mechanisms extend beyond cholinesterase inhibition (e.g., Hup A), encompassing distinct pathways. Matrine improves cognitive deficits by inhibiting Aβ aggregation and blocking the RAGE/Aβ axis [174], while evodiamine activates the PI3K/AKT/GSK3β pathway to ameliorate cognitive impairment [175]. Additionally, certain alkaloids display multi-target effects, such as reducing tau hyperphosphorylation and Aβ aggregation, highlighting their potential in drug development for NDs [176]. Therefore, future breakthroughs in preventing and treating NDs are anticipated by further exploration of the TCM treasure trove, gradually forming a comprehensive multi-channel and multi-target treatment strategy.

Compared to conventional administration routes, IN is gradually becoming a preferred method for encephalopathy research due to rapid onset, ease of use, no first-pass effect, fewer side effects, and effective BBB circumvention. However, IN still poses challenges, including limited nasal cavity volume and drug degradation. Nasal delivery of natural products is particularly challenging due to complex structures, poor druggability, and unfavorable physicochemical properties, hindering optimal efficacy. Moreover, IN of natural product-derived therapeutics may pose significant safety risks, necessitating comprehensive preclinical evaluations, particularly regarding dose-dependent mucosal toxicity and systemic bioaccumulation. For instance, preclinical studies have indicated that high-dose intranasal resveratrol induces local adverse effects, such as nasal mucosa irritation and neutrophilic inflammation, primarily mediated by activation of transient receptor potential channels. These local reactions may be exacerbated by systemic complications—including nausea, diarrhea, and hypotension—resulting from systemic distribution following trans-epithelial absorption [177]. Similarly, eucalyptus oil (primarily containing 1,8-cineole), widely used for symptomatic relief of nasal congestion, has been reported to induce irritation and pruritus in nasal and palatine mucosa following prolonged, high-dose nasal administration. Excessive use of eucalyptus oil nasal preparations may also trigger gastrointestinal symptoms such as heartburn and reflux, and lead to CNS toxicities, including headaches and dizziness, due to BBB penetration [178]. Nevertheless, due to the unique absorption environment and notable advantages of nasal administration, various carriers and modification strategies have been explored to improve nasal delivery of natural products. This review categorizes nasally administered natural products into six structural classes, summarizing common characteristics of carriers and modification methods employed for efficient nasal-brain delivery of these natural products.

For example, we found that: (I) Phenolic and phenolic acid compounds contain phenolic hydroxyl substitutions, causing structural instability and susceptibility to water, temperature, light, enzymes, and pH. Interestingly, phenolic hydroxyl groups are the key active groups for phenolic acid compounds to exert their antioxidant effects [179]. Studies have shown that phenolic hydroxyl groups on benzene rings are critical for inhibiting cholinesterase activity and providing antioxidant properties [180]. Therefore, protecting phenolic hydroxyl groups and enhancing the structural stability and antioxidant properties of phenolic acids represent significant challenges limiting their application. Some researchers have found that performance can be enhanced by modifying the phenolic ring structure. For instance, introducing electron-donating groups into phenolic compounds potentially improves their antioxidant capacity. Esterification could enhance antioxidant activity and thermal stability, while glycosylation may improve water solubility of phenolic acids [181]. Associating phenolic acids with chitosan is also a feasible strategy. Due to its strong mucosal adhesion and biocompatibility, chitosan could be employed as a penetration enhancer to facilitate phenolic acid absorption [182]. Selecting suitable carriers represents another critical step in enhancing the structural stability of phenols and phenolic acids. For instance, SLNs and NLCs, commonly used for intranasal delivery, effectively encapsulate phenols and phenolic acids, protecting active groups such as phenolic hydroxyls from environmental deterioration. Furthermore, surface functional modification of these nanocarriers could enhance specific brain targeting. For example, positively charged nanoparticles exhibit the highest brain uptake rate after IN [183]. Two principal factors explain this: the nasal mucus layer is negatively charged, prolonging the retention of positively charged carriers at the absorption site due to electrostatic interaction; additionally, positively charged nanoparticles effectively interact with negatively charged brain microvascular endothelial cells when traversing the BBB, promoting brain accumulation. (II) Glycoside compounds are often difficult to absorb due to their complex structures and high molecular weights. Although glycoside groups improve solubility, their high molecular weight, polar specific surface area, and strong hydrogen bonding limit glycoside penetration across cell membranes [184]. Spatial hindrance prevents receptor binding and cellular entry, generally decreasing the anti-inflammatory activity of glycoside compounds [185]. Introducing lipophilic groups to modify the glycoside structure or removing some hydrophilic groups while maintaining the stability of the pharmacophore may improve membrane permeability. For example, lipophilic prodrugs release hydrophilic parent molecules upon administration, exhibiting enhanced bioavailability and pharmacological activity compared to monomers [186]. In addition, employing lipophilic nanocarriers such as liposomes, which possess favorable biocompatibility, or in situ hydrogels with strong adhesive properties could enhance the transmembrane transport capability of glycosides and prolong their retention at absorption sites. This approach represents a promising drug delivery strategy [187]. (III) Flavonoids, as natural organic compounds exhibiting extensive pharmacological activity, face clinical challenges due to their poor water solubility, low bioavailability, and instability in vivo. Phospholipid complexes, being non-toxic and amphiphilic endogenous substances, can bind to one or more natural products via hydrogen bonding and electrostatic interactions, thereby enhancing the solubility and permeability of water-insoluble compounds [188]. Consequently, designing flavonoid-phospholipid complexes or employing phospholipid-based drug delivery systems such as liposomes may significantly enhance flavonoid bioavailability. In addition, flavonoids typically contain multiple phenolic hydroxyl groups, which can act as proton acceptors and donors, thus forming hydrogen bonds with eutectic ligands. Employing a eutectic strategy could effectively enhance their pharmacological efficacy and improve numerous physicochemical properties such as solubility, dissolution rate, stability, and melting point without altering their chemical structure [189]. (IV) Alkaloids, the primary pharmacologically active components of many herbal medicinal products, often suffer from low solubility, poor stability, short half-life, and limited oral bioavailability. In addition to their inherent physicochemical limitations, side effects are a significant concern for their clinical application. Numerous alkaloids exhibit potent biological activities at low concentrations; however, improper usage could lead to toxic side effects [190]. These alkaloids were both “active” and “toxic” natural products; thus, the safety of related natural compounds warrants special consideration. Notably, the positively charged quaternary ammonium ions present in alkaloids can mediate electrostatic interactions by attracting negatively charged groups nearby, thereby facilitating self-assembly. Through this process, toxic groups within the molecules may rearrange and interact differently, potentially reducing toxicity by forming supramolecular structures [191]. Furthermore, carriers such as liposomes, nanoparticles, gels, and solid lipid nanoparticles can facilitate alkaloid delivery while minimizing adverse reactions. Most of the above-mentioned carriers exhibit favorable sustained-release properties, prolonging drug retention at therapeutic sites and enabling gradual, continuous drug release, effectively avoiding drug accumulation and cumulative toxicity resulting from high-frequency administration [192]. (V) Terpenoids, derived from diverse sources and abundant in medicinal plants, are generally recognized as safe and non-irritating, enabling their extensive use as penetration enhancers in dermal and mucosal delivery. However, terpenoids’ limited lipophilicity, poor absorption capacity, low stability, and high molecular weight impede their permeability through lipid membranes, resulting in decreased bioavailability and activity. Studies have demonstrated that encapsulating terpenoids within lipid nanocarriers can enhance their lipid solubility and prevent chemical degradation and volatilization due to environmental factors. Furthermore, nanostructured lipid systems facilitate controlled drug release and enable bioactive compounds to cross biological barriers, positioning them as optimal candidates for local therapeutic applications [193].

The rapid evolution of advanced drug delivery systems has significantly enhanced the potential of IN for treating NDs. When selecting nanocarriers for CNS delivery, critical evaluation criteria include BBB penetration efficiency, drug-loading capacity, controlled-release performance, and biocompatibility/immunogenicity profiles (Supplementary Table S1) [194,195,196]. Among current platforms, exosomes exhibit unique advantages due to their endogenous origin, including intrinsic targeting capability, superior BBB permeability, and low immunogenicity, particularly when derived from autologous sources, making them suitable for chronic therapeutic regimens [197]. However, exosomes face limitations in drug encapsulation efficiency because of constraints imposed by their bilayer membranes. In contrast, synthetic nanoparticles such as polymeric nanoparticles (e.g., PLGA-based systems) demonstrate exceptional drug-loading capacities owing to their hydrophobic cores and tunable polymer matrices, while liposomes offer dual-phase release kinetics (burst and sustained release) through engineered phospholipid compositions [198,199]. Similarly, nanoemulsions achieve optimized drug solubilization and release profiles via adjustable structural parameters (such as oil-phase composition, interfacial tension, and particle size) [200]. In addition to the carriers and modification methods previously mentioned, two novel, promising approaches for nasal-brain delivery of natural products have been identified: (I) Microneedle-mediated nasal-brain drug delivery, which utilizes microneedles as an active, minimally invasive method to overcome physiological barriers and achieve precise drug administration [201]. Secondly, (II) self-assembling delivery systems for natural products [202]. Driven by non-covalent interactions, natural products form various aggregates or self-assembled particles, such as nanoparticles, micelles, and gels, which promote drug efficacy through self-processing, self-assembly, and self-repair mechanisms. While most natural product-based systems remain in preclinical development, several polymeric and lipid-based nanoparticles (e.g., paclitaxel-loaded PLGA nanoparticles) have advanced to Phase II clinical trials, demonstrating acceptable safety profiles. Key translational challenges include molecular size constraints, mucosal clearance, nanocarrier toxicity, compound instability, and regulatory complexities. Future innovations may integrate multidisciplinary approaches, such as: hybrid systems (e.g., exosome-coated liposomes) combining endogenous targeting with synthetic versatility; stimuli-responsive carriers (e.g., pH- or enzyme-sensitive nanovehicles) for lesion-specific drug activation; and combinatorial therapies co-delivering natural products with biologics (e.g., anti-Aβ antibodies) for multitarget modulation. In summary, the increasing demand for therapeutic agents to treat NDs urgently necessitates thorough investigation into novel delivery systems and accelerated development of drug resources.

## Figures and Tables

**Table 1:** Etiology, age of onset, and characteristic clinical manifestations of neurodegenerative diseases.

Disease	Etiology	Age of Onset	Characteristic Clinical Manifestations	Reference
Alzheimer’s disease	Genetic factors: APP, PSEN1, and PSEN2 gene mutations; the APOE ε4 allele increases the risk. Environmental factors: age, hypertension, diabetes, head trauma, etc.	Predominantly occurs after 65 years (late-onset), with rare presentations before 65 (early-onset).	Cognitive manifestations: memory impairment (especially recent memory), language dysfunction, and disorientation. Behavioral symptoms: depression, anxiety, apathy, and hallucinations. Functional decline: decreased ability to perform activities of daily living.	[4]
Parkinson’s disease	Genetic factors: LRRK2, PARK2, SNCA, and other gene mutations. Environmental factors: exposure to pesticides, heavy metals, etc. Neuropathology: loss of dopaminergic neurons in substantia nigra.	Predominantly occurs at 50–70 years, with rare presentations before 50 (early-onset).	Movement symptoms: resting tremor, rigidity, bradykinesia, and postural imbalance. Non-motor symptoms: hyposmia, constipation, depression, and sleep disturbances.	[5]
Huntington’s disease	Genetic factors: CAG trinucleotide repeat amplification (>40 times) in the HTT gene.	Predominantly occurs at 30–50 years, with rare presentations before 20 (early-onset) or after 50 (late-onset).	Motor symptoms: choreiform movements, dystonia, and gait abnormalities. Cognitive symptoms: executive dysfunction and memory loss. Psychiatric symptoms: depression, anxiety, irritability, and psychotic symptoms.	[6]
Amyotrophic lateral sclerosis	Genetic factors: SOD1, C9ORF72, TARDBP, and other gene mutations. Environmental factors: smoking, heavy metal exposure, strenuous exercise, etc.	Predominantly occurs at 40–70 years, with rare presentations before 40 (early-onset).	Motor symptoms: muscle weakness, neurogenic atrophy (typically from the extremities or medulla oblongata), and fasciculation. Function decline: dysphagia, dysarthria, and dyspnea. Cognitive symptoms: Co-occurring frontotemporal dementia in subset of patients	[7]
Spinal muscular atrophy	Hereditary factors: Deletion or mutation of the SMN1 gene, resulting in a deficiency of the motor neuron survival protein (SMN).	Infants (0–6 months), children (6 months–18 years), and adults (after 18 years).	Motor symptoms: progressive muscle weakness and muscle atrophy (proximal heavier than distal). Function decline: dyspnea and dysphagia (severe type). Others: Scoliosis and joint contracture.	[8]
Hereditary spastic paraplegia	Genetic factors: SPAST, ATL1, REEP1, and other gene mutations. Pathology: degeneration of corticospinal tract.	Predominantly occurs 10–40 years, with rare presentations before 10 or after 40.	Movement symptoms: spastic weakness of lower limbs and abnormal gait. Other symptoms: bladder dysfunction and mild cognitive impairment (complex hereditary spastic paraplegia).	[9]
Multiple sclerosis	Genetic factors: HLA-DRB1 gene polymorphism. Environmental factors: viral infection, vitamin D deficiency, smoking, etc. -Pathology: demyelination and inflammation of the central nervous system.	Predominantly occurs 20–40 years, with rare presentations before 20 or after 40.	Motor symptoms: limb weakness, ataxia, and spasticity. Sensory symptoms: numbness, tingling, and decreased vision (optic neuritis). Other symptoms: fatigue, cognitive impairment, and bladder dysfunction.	[10]

## Data Availability

No data was used for the research described in the article.

## Supplementary Materials

The following supporting information can be downloaded at: https://www.mdpi.com/article/10.3390/pharmaceutics17050675/s1, Supplementary Table S1. Comparison of drug carriers for central nervous system applications.
