# Supplementary material for: Application of Natural Products in Neurodegenerative Diseases by Intranasal Administration: A Review
Source: Pharmaceutics. 2025 May 20;17(5):675. doi: 10.3390/pharmaceutics17050675 (PMC12114702; doi:10.3390/pharmaceutics17050675)
Supplement: Supplementary file 1 [file pharmaceutics-17-00675-s001.zip › pharmaceutics-3631032-supplementary.pdf]

Supplementary Table S1. Comparison of drug carriers for central nervous system applications

Delivery System	BBB Penetration Mechanism	Payload Type	Release Kinetics	Biocompatibility	Immunogenicity Risk	Clinical Stage	Key Challenges	References
In Situ Gels	Nasal administration with gelation to prolong retention, bypassing BBB via olfactory/trigeminal pathways	Small molecules, peptides	Ion/pH-responsive gelation; sustained release (hours to days)	Biodegradable materials (e.g., gellan gum, chitosan): favorable	Low; high ionic strength may irritate mucosa	Early-phase trials (e.g., nasal Alzheimer's drugs)	Dose precision, mucosal irritation	[194]
Artificial Vesicles	Synthetic exosome mimics (e.g., membrane-coated NPs) with customizable ligands for targeted delivery	Nucleic acids, hydrophobic drugs	Microenvironment-dependent triggered release	Biomimetic membranes (e.g., erythrocyte-derived): low immunogenicity	Minimal (autologous membrane sources)	Preclinical; no CNS-targeted clinical trials	Complex fabrication, in vivo tracking limitations	[195]
Solid Lipid NPs	Passive diffusion via small size (<200 nm) or active targeting (e.g., ApE peptide modification)	Lipophilic drugs, antioxidants	Lipid matrix-mediated sustained release (1–7 days)	Natural lipids (e.g., triglycerides): safe; potential lipid metabolism interference	Low, but hepatotoxicity risk with chronic use	Phase I/II trials (e.g., carmustine SLNs for glioma)	Low drug loading, oxidative instability	[196]
Exosomes	Native membrane proteins (e.g., LAMP2B) mediate BBB traversal via endogenous cell communication pathways	siRNA, miRNA, small hydrophilic drugs	Uncontrolled release via membrane fusion/endocytosis	Autologous: low toxicity; allogeneic: requires purification	Low (immune evasion by natural membrane proteins)	Early-phase trials (I/II, e.g., MSC exosomes for stroke)	Standardized production, low drug-loading efficiency	[197]
Polymeric Nanoparticles	Surface modification with targeting ligands (e.g., transferrin receptor antibodies) or PEGylation to enhance receptor-/adsorption-mediated transport	Hydrophobic drugs, nucleic acids, proteins	Polymer degradation-dependent (e.g., PLGA: days to weeks)	Material-dependent (PLGA/PEG: high; PEI: cytotoxic)	Synthetic materials may trigger inflammation; mitigated by surface engineering	Phase II/III trials (e.g., brain-targeted paclitaxel-PLGA)	Scale-up consistency, long-term toxicity evaluation	[198]

Supplementary Table S1 (continued)

Delivery System	BBB Penetration Mechanism	Payload Type	Release Kinetics	Biocompatibility	Immunogenicity Risk	Clinical Stage	Key Challenges	References
Liposomes	PEGylated long-circulating design or Angiopep-2-modified targeting to LRP1 receptors	Hydrophilic/hydrophobic drugs, gene therapies	Biphasic release (burst + sustained; hours to days)	Phospholipid metabolizable; high cholesterol may destabilize membranes	Low (classic liposomes); cationic variants: higher toxicity	Marketed (non-CNS); CNS applications in Phase II	Accelerated blood clearance (PEG-related)	[199]
Nanoemulsions	Enhanced lipophilicity for passive diffusion or transient BBB opening (e.g., ultrasound/mannitol co-administration)	Lipophilic drugs, essential oils	Rapid release (hours to 1 day)	Natural lipids (e.g., soy lecithin): generally safe	Low, but surfactants may cause mucosal irritation	Phase II for nasal delivery; limited systemic CNS applications	Poor stability, reliance on physical BBB disruption	[200]
